# Composite mathematical modeling of calcium signaling behind neuronal cell death in Alzheimer’s disease

**DOI:** 10.1186/s12918-018-0529-2

**Published:** 2018-04-11

**Authors:** Bobby Ranjan, Ket Hing Chong, Jie Zheng

**Affiliations:** 1 Biomedical Informatics Lab, School of Computer Science and Engineering, Nanyang Technological University, 50 Nanyang Avenue, Singapore, 639798 Singapore; 2 Genome Institute of Singapore, Biopolis, Singapore, 138672 Singapore; 3 Complexity Institute, Nanyang Technological University, Nanyang Drive, Singapore, 637723 Singapore

**Keywords:** Calcium signaling, Neuronal cell death, Alzheimer’s disease, Mathematical modeling

## Abstract

**Background:**

Alzheimer’s disease (AD) is a progressive neurological disorder, recognized as the most common cause of dementia affecting people aged 65 and above. AD is characterized by an increase in amyloid metabolism, and by the misfolding and deposition of *β*-amyloid oligomers in and around neurons in the brain. These processes remodel the calcium signaling mechanism in neurons, leading to cell death via apoptosis. Despite accumulating knowledge about the biological processes underlying AD, mathematical models to date are restricted to depicting only a small portion of the pathology.

**Results:**

Here, we integrated multiple mathematical models to analyze and understand the relationship among amyloid depositions, calcium signaling and mitochondrial permeability transition pore (PTP) related cell apoptosis in AD. The model was used to simulate calcium dynamics in the absence and presence of AD. In the absence of AD, i.e. without *β*-amyloid deposition, mitochondrial and cytosolic calcium level remains in the low resting concentration. However, our *in silico* simulation of the presence of AD with the *β*-amyloid deposition, shows an increase in the entry of calcium ions into the cell and dysregulation of Ca ^2+^ channel receptors on the Endoplasmic Reticulum. This composite model enabled us to make simulation that is not possible to measure experimentally.

**Conclusions:**

Our mathematical model depicting the mechanisms affecting calcium signaling in neurons can help understand AD at the systems level and has potential for diagnostic and therapeutic applications.

**Electronic supplementary material:**

The online version of this article (10.1186/s12918-018-0529-2) contains supplementary material, which is available to authorized users.

## Background

Alzheimer’s disease (AD) is characterized by the deposition of *β*-amyloid (A *β*) oligomers in and around neurons in the brain accompanied by dysfunctional neuronal calcium homeostasis. Autophagy is generally an efficient mechanism for removing amyloids. During the onset of AD, autophagy is increased but the transfer of autophagic vesicles to the lysosomes is blocked [1]. This may contribute to the accumulation of amyloids. There is increasing evidence to support the hypothesis that A *β* induces an up-regulation of intracellular Ca ^2+^ and leads to AD. Multiple studies on AD mouse models have shown that Ca ^2+^ dysregulation leads to increased Ca ^2+^ entry into the cytoplasm resulting in neuronal cell death and AD [2, 3].

The observations about the effect of A- *β* oligomers on neuronal calcium signaling led to the formulation of the calcium hypothesis of AD [4]. The basic argument behind the hypothesis is that the activation of the amyloidogenic pathway results in a remodeling of the neuronal calcium signaling pathway. The up-regulation of Ca ^2+^ distorts the normal neuronal Ca ^2+^ signaling by increasing the amount of Ca ^2+^ being taken up by the mitochondria. A sustained increase in the mitochondrial Ca ^2+^ may activate the mitochondria to initiate the intrinsic pathway of Ca ^2+^-induced apoptosis, as described in the calcium hypothesis of Alzheimer’s disease [4].

According to Berridge, the increased output of *Ca*^2+^ due to the hypersensitivity of the *Ca*^2+^ signaling system may activate the mitochondria to initiate the intrinsic pathway of *Ca*^2+^-induced apoptosis by opening up the mitochondrial permeability transition pore (PTP), causing collapse of the mitochondrial membrane potential and releasing cytochrome c and other factors that activate the caspase cascade responsible for apoptosis.

Ichas and Mazat [5] demonstrated that the mitochondrial PTP operates at the crossroads of 2 distinct physiological pathways i.e. the *Ca*^2+^ signaling network during the life of the cell and the effector phase of the apoptotic cascade during *Ca*^2+^-dependent cell death. It has 2 open conformations correspondingly. The low-conductance state, which allows the diffusion of small ions like *Ca*^2+^, is pH-operated, promoting spontaneous closure of the channel. A high-conductance state, which allows the unselective diffusion of big molecules, stabilizes the channel in open conformation [5].

Mitochondria in open high-conductance state can no longer maintain a proton gradient, and thus cannot sustain oxidative phosphorylation, resulting in an arrest of aerobic ATP synthesis (necrotic cell death). This also results in an oncotic imbalance in the mitochondria causing it to swell up. The cristae formed by the inner membrane unfold, leading to rupture of the outer membrane that brings into direct communication the former intermembrane space and the extra-mitochondrial medium. Soluble components like cytochrome c and Apoptosis Inducing Factor (AIF), which are normally trapped in the intermembrane space, are released into the cytosol, thereby inducing cell apoptosis [5].

Thul [6] described the use of Ordinary Differential Equations (ODEs) to model intracellular *Ca*^2+^ oscillations, assuming intracellular *Ca*^2+^ concentration to be spatially homogeneous. The use of ODEs is widespread among modelers because: (a) the study of ODEs is computationally well-supported, with a large body of techniques available to investigate ODEs in great detail, and (b) lack of sufficient experimental data to develop a spatially extended model.

In this paper the processes mentioned above have been modeled mathematically using ODEs to allow for quantitative understanding of the dynamics of neuronal cell death in AD. We achieved this by integrating three models: Fall-Keizer Model [7], Mitochondrial PTP Model [8], and Amyloid Metabolism Model [9]. These models are explained in detail in the next section. The results obtained are qualitatively consistent with all the three papers. To demonstrate the validity of our composite model, we tested two hypotheses proposed by these individual models:

1. When there is no abnormality in *β*-amyloid folding and deposition, the initiation of an action potential does not lead to long-term sustained oscillations (an extension of the result detailed in [7]). This will be explained further in the Results section.
2. When *β*-amyloid misfolding affects calcium signaling within the neuron, the action potential is prevented from dying down immediately (a more complex expression of the qualitative trend represented by [9]). As *β*-amyloid deposition increases with time, the rate of entry of calcium ions into the cell increases, thus allowing the calcium ion oscillations to continue by maintaining high calcium ion levels in the cytosol. As this rate of entry continues to increase, the cytosol and mitochondria attempt to release excess calcium ions to one another more frequently and in smaller amounts, thus resulting in smaller but more rapid oscillations.

We used time-course simulation to relate the occurrence of events to biological processes, thereby verifying our model. We also quantified certain findings from the time-course simulations, with special emphasis on the time taken for the PTP to open in high conductance state.

## Methods

**Fig. 1:**
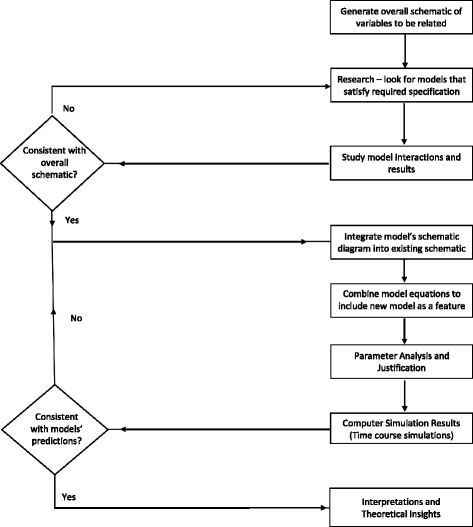
Flow chart of analyzing existing models and constructing one composite model

The process of building a composite model from individual models is depicted in the flow chart in Fig. 1. Our objective is that the composite model should not only satisfy the properties of the individual models but also help lead biologists using this model to additional insights regarding the processes described here. Specifically, for an in-depth study of calcium signaling we integrate key components from the three models to investigate the dynamics of calcium oscillations to neuronal cell death. The three models are explained in the following subsections.

**Fig. 2:**
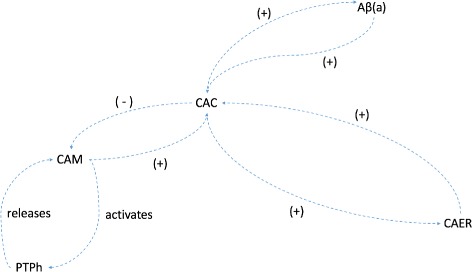
Composite Model Schematic relating Cytosolic Ca ^2+^ concentration (CAC), Mitochondrial Ca ^2+^ concentration (CAM), Endoplasmic Reticulum Ca ^2+^ concentration (CAER), *β*-amyloid (a) concentration and PTP high-conductance activation state (PTPh)

As depicted in the model schematic diagram (Fig. 2), the composite model consists of four main molecular species, namely Cytosolic Ca ^2+^ (CAC), Mitochondrial Ca ^2+^ (CAM), Endoplasmic Reticulum Ca ^2+^ (CAER) and beta-amyloid (A *β*). In addition, a node is included to represent the mitochondria in high-conductance state (PTPh).

### The Fall-Keizer model

The Fall-Keizer model is an integrated model depicting mitochondrial Ca ^2+^ handling and metabolic function. It integrates the Magnus-Keizer model [10, 11] and the De Young-Keizer model [12]. The Magnus-Keizer model is a comprehensive mitochondrial model with six proton transfer mechanisms that affect Ca ^2+^ signaling.

A key motivation for using the Fall-Keizer model was to improve the original Magnus-Keizer model, by modifications to the Ca ^2+^ uniporter so that the prediction of Ca ^2+^ signaling can be more accurate.

The inclusion of the De Young-Keizer model for inositol-1,4,5-triphosphate (IP3)-mediated Ca ^2+^ release along with appropriate scaling and provisions for accommodating different cell types allows a modeler to easily shape this model to his or her objective. Furthermore, the comprehensive nature and the modularity of the model that have been maintained by Fall and Keizer make this model an ideal choice for the purposes of this paper.

The model has over 12 variables interacting with each other to depict the Ca ^2+^ signaling in a cell. The basic mitochondrial function has been taken from the Magnus-Keizer model, with a modified mechanism for Ca ^2+^ uptake introduced by Fall and Keizer [7]. Furthermore, the modular nature of this model allows for addition of the PTP characteristics which were omitted in the Fall-Keizer model.

1 $$ {{\begin{aligned} \frac{dCAC}{dt}= \frac{f_{i}}{V_{c}\tau_{min}}\left[M\left(J_{Na,ex}^{Ca}-J_{uni}^{Ca}-J_{PTP}^{Ca}\right)-E\left(J_{serca}-J_{er,out}\right)\right] \end{aligned}}}  $$

Equation (1) was obtained from the Fall-Keizer model [7]. CAC is directly proportional to the total volume of mitochondria in the cell (*M*) multiplied by the rate of transfer of calcium ions into the cytosol from the mitochondria, which is dependent on the sodium-calcium ion exchanger, the calcium uniporter and the PTP. CAC is also directly proportional to the total volume of ER in the cell (*E*) multiplied by the rate of transfer of calcium ions into the cytosol from the ER, which is affected by the SERCA pump and the leakage of calcium ions from the ER.

Since our focus is on mitochondria-induced cell apoptosis, this model provides us with the appropriate foundation to build upon.

### The Mitochondrial PTP model

The model proposed by Oster et al. [8] provides a representation of the mitochondrial permeability transition pore (PTP) behavior. In our model, we have focused on the high conductance state of PTP. In its high-conductance conformation, PTP opening induces unselective solute fluxes that dissipate the concentration gradients of relatively big molecules. However, since most proteins remain trapped in the matrix, the resulting oncotic imbalance (at least in vitro) causes high amplitude swelling of the organelle. The subsequent unfolding of the inner membrane causes rupture of the outer membrane, which results in the release of soluble components (mainly cytochrome c and Apoptosis Inducing Factor (AIF)) that are normally located in the intermembrane space [13–15].

The transition of the pore to a high-conductance state requires prolonged levels of high mitochondrial Ca ^2+^. Once the pore opens to this state, it remains open, leading to cell death. The model proposed by Oster et al. [8] assumes that whether or not the pore enters the high-conductance state depends on a secondary slow process, which in turn depends on the overall mitochondrial Ca ^2+^ load.

### The Amyloid metabolism model

It is widely known that amyloids perturb Ca ^2+^ homeostasis, and *β*-amyloids perturb the balance between Ca ^2+^ entry in and extrusion out of the cytoplasm. In healthy neurons, these processes equilibrate, leading to a basal Ca ^2+^ level in the range of 50-100 nM [16]. Studies on the cortical neurons of AD stricken animals found a basal Ca ^2+^ level of around 250 nM, i.e. around twice that found in controls [17].

Based on these observations, we believe that the model proposed by Caluwe and Dupont [9] provides an accurate representation of the relationship between *β*-amyloid protein concentration and cytoplasmic calcium concentration. Their depiction of a positive feedback loop supports the increased basal Ca ^2+^ levels observed in cortical neurons with the accumulation of amyloids. Furthermore, research conducted on the production of the toxic oligomers found that Ca ^2+^ ions actually promote the synthesis of *β*-amyloids [18]. These results support the existence of a bistable state switch in the neurons due to the positive feedback loop between the amyloid concentration and the calcium ion concentration. Equation (2), describing the change in intracellular calcium concentration, has been taken from the Caluwe and Dupont model [9].

2 $$ \frac{dCAC}{dt}=V_{2}+K_{\beta}a^{m} - k_{2} * CAC  $$

This equation shows the effect of amyloids on intracellular calcium concentration. It was formulated based on the assumption that amyloids increase intracellular calcium concentration by increasing the permeability of plasma membranes. This assumption is also made in our model. Here, the rate at which calcium enters the cytoplasm and the rate of eliminating calcium ions from the cytoplasm are assumed to be constant. Furthermore, calcium ion concentration in the cytoplasm is assumed to have first order kinetics.

### Composite model

We used XPPAUT [19], an.ode model file as in Additional file 1 and MATLAB to plot our time-course simulations. Details of the composite model constructed based on the afore-mentioned three models are given below. A schematic diagram of the composite model is illustrated in Fig. 2. The parameters used in our composite model were obtained from the individual models.

The model equations for these molecular species are given below and the model parameters are given in Table 1. For the meaning of the rate constants and other parameters readers are to refer to Tables 1, 2 and 3. In an attempt to extend the models described previously and make them more comprehensive, we have combined components and effects that act on the same ion concentration. For instance, here, we used the components affecting CAC in Eqs. (1) and (2) to create the composite equation shown in Eq. (3):

3 $$ {{\begin{aligned} \frac{dCAC}{dt}\,=\, \frac{f_{i}}{V_{c}\tau_{min}}\!\left[\!M\left(J_{Na,ex}^{Ca}\,-\,J_{uni}^{Ca}-J_{PTP}^{Ca}\right)\,-\,E\left(J_{serca}\,-\,J_{er,out}\right)\right] \,+\, k_{\beta}a^{m} \end{aligned}}}  $$

**Table 1:** List of parameters used in our model with their values and biological significance [7–9]

Parameters	Value	Biological significance
*V*	1 *ml*	Total volume
*uMmM*	1000	Converts *μ*M to mM
*τ*_*min*_	60	Converts minutes to seconds
*p*_*cytosol*_	0.5	Proportion of volume occupied my cytosol
*d*_*cytosol*_	75 *mg*/*ml*	Density of cytosolic protein mg/ml
*p*_*mito*_	0.05	Proportion of volume occupied my mitochondria
*d*_*mito*_	1000 *mg*/*ml*	Density of mitochondrial protein mg/ml
*p*_*er*_	0.1	Proportion of volume occupied my ER
*d*_*er*_	1000 *mg*/*ml*	Density of ER protein mg/ml
*c*_*mito*_	0.0725 $\frac {nmol}{mV*mg}$	Mitochondrial calcium concentration
*ρ*_*uni*_	300 $\frac {nmol}{mg*min}$	Maximum rate of transport through mitochondrial uniporter
$\rho _{Na}^{Ca} $	3 $\frac {nmol}{mg*min}$	Maximum rate of transport through *Na*^+^/*Ca*^2+^ exchanger
*ρ*_*res*_	0.4	Mitochondrial respiration co-efficient
*ρ*_*F*_1	0.7 $\frac {nmol}{mg*min} $	Mitochondria - Fo/F1 ATPase
[*P*_*i*_]_*m*_	20 *mM*	Concentration of free phosphates
*ρ*_*leak*_	0.2 $\frac {nmol}{mg*min} $	Mitochondrial membrane proton leak
*J*_*red*,*basal*_	20 $\frac {nmol}{mg*min}$	NADH reduction rate
*J*_*max*,*ANT*_	900 $\frac {nmol}{mg*min}$	ATP/ADP antiport flux
*glc*	1 *mM*	Glucose concentration in cytosol
*J*_*hyd*,*max*_	30.1 *mM*	Cytosol hydrolysis of ATP
*V*_*IP*3_	3000 *μM*	*IP*_3_ receptor volume
*V*_*leak*_	0.1	proportion of leakage from *IP*_3_ receptor
*J*_*leak*_	0.1	ER leak
*d*_*IP*3_	0.25 *μM*	*IP*_3_ receptor sensitivity
*d*_*ACT*_	1 *μM*	*IP*_3_ receptor *Ca*^2+^ activation constant
*d*_*INH*_	1.4 *μM*	*IP*_3_ receptor *Ca*^2+^ inhibition constant
*τ*	4 *s*	*IP*_3_ receptor inhibitory time constant
*V*_*serca*_	110 $\frac {nmol}{mg*min}$	SERCA pump flux
*k*_*serca*_	0.4 *μM*	SERCA pump *Ca*^2+^ sensitivity
*V*_1_	0.0065 *nM*/*s*	Constant rate of *β*-amyloid synthesis
*V*_*α*_	0.05 *nM*/*s*	Maximal rate of *β*-amyloid synthesis
*K*_*α*_	120 *nM*	Half-saturation constant
*k*_*β*_	0.2 *nM*^3^/*s*	Rate constant of increased *Ca*^2+^ entry
*K*_1_	0.01 *s*^−1^	Rate constant of *β*-amyloid elimination
*n*	2	Hill coefficient for activation of *β*-amyloid synthesis
*m*	4	Cooperativity coefficient
*f*_*m*_	0.0003	Mitochondrial *Ca*^2+^ buffering coefficient
*f*_*i*_	0.01	Cytosolic *Ca*^2+^ buffering coefficient

**Table 2:** List of parameters used in *IP*_3_ step function for *IP*_3_-mediated calcium oscillations [7–9]

Parameters	Value	Biological significance
*b* *a* *s* *e* *l* *i* *n* *e*	0.3 *μM*	Base concentration
*a* *m* *p* *l* *i* *t* *u* *d* *e*	0.3 *μM*	Oscillation amplitude
*i* *n* *i* *t*	10 *ms*	Initial time
*d* *u* *r* *a* *t* *i* *o* *n*	100 *ms*	Duration of oscillations

**Table 3:** List of parameters used in PTP Integration for PTPh and PTPl states [7–9]

Parameters	Value	Biological significance
*C* *A* *M* ^∗^	4 *μM*	Threshold Mitochondrial *Ca*^2+^
*y* ^∗^	0.8	Secondary process threshold
$f_{H_{M}} $	1.28 x 10 ^−6^	Fast buffering constant for protons in mitochondria
*p*_1_	0.022	Parameter *p*_1_
*p*_2_	0.0001	Parameter *p*_2_
*p*_3_	0.0231	Parameter *p*_3_
*p*_4_	0.0001	Parameter *p*_4_
*a* *m* *p*_*τ*_	26000	Amplitude for time constant
*p*_6_	0.001	Parameter *p*_6_
$perm_{l}^{H}$	3.0	PTP Permeability to protons
*p* *e* *r* *m*_*Ca*_	0.4	PTP Permeability to calcium ions
*postptp*	2	PTP opening indication constant

This equation shows the effect of amyloid concentration inducing calcium ion entry into the cell by taking into account its effect of increasing plasma membrane permeability. The effect of amyloid deposition on cytosolic calcium concentration is quantified as the rate of increase of the *Ca*^2+^ entry multiplied by the amyloid concentration raised to its cooperativity coefficient.

4 $$ \frac{dCAM}{dt}=\frac{f_{m}M}{V_{m}\tau_{min}}\left(J_{uni}^{Ca}-J_{Na,ex}^{Ca}+J_{PTP}^{Ca}\right)  $$

The dynamics of mitochondrial calcium ion concentration is shown in Eq. (4), obtained from Oster et al. [8]. It increases with the calcium ion influx through the uniporter and PTP, and it decreases with the calcium ion efflux through the Na ^+^-Ca ^2+^ ion exchanger.

5 $$ \frac{dCAER}{dt}=\frac{f_{i}E}{V_{e}\tau_{min}}\left(J_{serca}-J_{er,out}\right)  $$

ER calcium ion concentration varies with influx through the SERCA (Sarcoplasmic/Endoplasmic Reticulum Calcium ATPase) pump and efflux by leakage of calcium ions, obtained from Fall and Keizer (2001) [7].

6 $$ \frac{da}{dt}=V_{1}+V\alpha\left(\frac{1}{1+\frac{K_{\alpha}^{n}}{CAC^{n}}}\right)-K_{1}a  $$

Equation (6) focuses on the rate of change of amyloid concentration (a) in the cell. Like Caluwe and Dupont [9], we picked amyloid concentration as a generic quantity, encompassing intracellular and extracellular amyloid concentrations, along with amyloid compounds of different lengths and oligomerization states. The rate of amyloid synthesis is assumed to be a constant. The increase of the cytosolic calcium concentration increases the amyloid concentration as well, according to Caluwe and Dupont [9]. Moreover, the increased amyloid concentration negatively affects its own increase in gradient, allowing some form of regulation of amyloids in spite of cytosolic calcium ion concentration.

7 $$ \frac{dPTP_{h}}{dt}=\frac{PTP_{h}^{\infty}-PTP_{h}}{\tau_{h}}  $$

Equation (7) is derived from Oster et al. [8]. It depicts the mitochondrial PTP in its high conductance state as a binary state controlled by a Heaviside function. This depends on a secondary slow process, which is also a Heaviside function function that depends on a mitochondrial calcium ion concentration crossing a particular threshold. This is the time constant used to provide a time lag in simulating the actual switching of the PTP state. Tables 4, 5, 6, 7, 8, 9, 10, 11, 12, 13, 14, 15, 16 and 17 contain the equations, parameters and initial conditions from other models that have been used in this model.

**Table 4:** List of model ODEs from [7–9]

Equation	Biological significance
*dNADH*_*m*_/*dt*=(*J*_*red*_−*J*_*o*_)∗*M*/(*uMmM*∗*V*_*m*_∗*minute*)	ODE for mitochondrial NADH concentration change (mM/s)
*dADP*_*m*_/*dt*=(*J*_*ANT*_−*J*_*p*,*TCA*_−*J*_*p*,*F*1_)∗*M*/(*uMmM*∗*V*_*m*_∗*minute*)	ODE for mitochondrial ADP concentration change (mM/s)
*dADP*_*i*_/*dt*=(−*J*_*ANT*_∗*M*+(*J*_*hyd*_−*J*_*p*,*gly*_)∗*C*)/(*uMmM*∗*V*_*c*_∗*minute*)	ODE for cytosolic ADP concentration change (mM/s)
$dPSI/dt= -\left (-J^{H}_{res} + J_{H,F1} + J_{ANT} +J^{H}_{PTP} +J^{H}_{L} +2*J_{uni} +2 * J^{Ca}_{PTP}\right) * M/\left (c_{mito} * minute \right)$	ODE for mitochondrial inner membrane voltage
*dh*/*dt*=(*d*_*INH*_−(*CAC*+*d*_*INH*_)∗*h*)/*τ*	ODE for change in percentage of closed channels

**Table 5:** List of initial conditions [7–9]

Parameters	Value	Biological significance
*PSI*(0)	164 *mV*	Base potential at time 0
*CAM*(0)	0.05 *μM*	Mitochondrial *Ca*^2+^ concentration at time 0
*CAC*(0)	0.05 *μM*	Cytosolic *Ca*^2+^ concentration at time 0
*CAER*(0)	11 *μM*	ER *Ca*^2+^ concentration at time 0
*ADP*_*m*_(0)	4.46 *mM*	Mitochondrial ADP concentration at time 0
*ADP*_*i*_(0)	0.028 *mM*	Cytosolic ADP concentration at time 0
*NADH*_*m*_(0)	0.16 *mM*	Mitochondrial NADH concentration at time 0
*h*(0)	95%	Percentage of closed channels at time 0
*a*(0)	0 *mM*	*β*-amyloid concentration at time 0
*y*(0)	0	Secondary slow process involved in PTP opening at time 0
*PTP*_*h*_(0)	0	PTP closed at time 0
*PTP*_*l*_(0)	0	PTP closed at time 0

**Table 6:** List of model equations used in calculation of protein amounts [7–9]

Equation	Biological significance
*M*=*V*∗*p*_*mito*_∗*d*_*mito*_	Calculation of mitochondrial protein amount
*C*=*V*∗*p*_*cytosol*_∗*d*_*cytosol*_	Calculation of cytosolic protein amount
*E*=*V*∗*p*_*er*_∗*d*_*er*_	Calculation of ER protein amount

**Table 7:** List of model equations used in calculation of compartment volumes [7–9]

Equation	Biological significance
*V*_*m*_=(*V*∗*p*_*mito*_)	Calculation of mitochondrial compartment volumes
*V*_*c*_=(*V*∗*p*_*cytosol*_)	Calculation of cytosolic compartment volumes
*V*_*e*_=(*V*∗*p*_*er*_)	Calculation of ER compartment volumes

**Table 8:** List of model equations used in nucleotide conversion/conservation relations (obtained from [10, 11])

Equation	Biological significance
*ATP*_*m*_=(12∗*d*_*mito*_/*uMmM*)−*ADP*_*m*_	Mitochondrial ATP concentration
*NAD*=(8∗*d*_*mito*_/*uMmM*)−*NADHM*	Mitochondrial NAD concentration

**Table 9:** List of model equations used in calculation of proportion of free nucleotides (obtained from [11])

Equation	Biological significance
*ADP*_*mf*_=0.8∗*ADP*_*m*_	Unbound mitochondrial ADP concentration
*ADP*_*if*_=0.3∗*ADP*_*i*_	Unbound cytosolic ADP concentration

**Table 10:** List of model equations used in calculation of proportion of charged, free nucleotides (obtained from [11])

Equation	Biological significance
[*ADP*^3−^]_*m*_=0.45∗*ADP*_*mf*_	Unbound, 3- charged mitochondrial ADP concentration
[*ADP*^3−^]_*i*_=0.45∗*ADP*_*if*_	Unbound, 3- charged cytosolic ADP concentration
[*MgADP*^−^]_*i*_=0.55∗*ADP*_*if*_	Unbound, 1- charged cytosolic *MgADP*^−^ concentration
[*ATP*^4−^]_*i*_=0.05∗*ATP*_*i*_	Unbound, 4- charged cytosolic ATP concentration
[*ATP*^4−^]_*m*_=0.05∗*ATP*_*m*_	Unbound, 4- charged mitochondrial ATP concentration

**Table 11:** List of model equations used in mitochondrial *Ca*^2+^ handling (obtained from [10])

Equation	Biological significance
Mitochondrial uniporter equations	
*MWC*_*num*_=(*CAC*/6)∗((1+(*CAC*/6))^3^)	MWC numerator
*MWC*_*denom*_=((1+(*CAC*/6))^4^)+(50/((1+(*CAC*/0.38))^2.8^))	MWC denominator
*MWC*=*MWC*_*num*_/*MWC*_*denom*_	MWC fraction value
$ V^{D}_{uni} = \left (PSI - 91\right)/13.35 $	Uniporter potential exponent
$ J_{uni} = \left (\rho _{uni} * V^{D}_{uni} * \left (MWC - CAM * exp\left (-V^{D}_{uni}\right)\right)/\left (1 - exp\left (-V^{D}_{uni}\right)\right)\right) * \left (1-PTP_{h}\right) $	Rate of transport through uniporter considering PTP in high conductance state
*Na*^+^/*Ca*^2+^ exchanger equations	
$ V^{D}_{nc} = exp\left (\left (PSI - 91\right)/53.4\right) $	*Na*^+^/*Ca*^2+^ exchanger potential generated
$ J_{nc} = \left (\rho _{nc} * V^{D}_{nc} * \left (1/\left (1 + \left (9.4/30\right)**2\right)\right) * \left (1/\left (1 + \left (0.003 * Dmito/CAM\right)\right)\right)\right) * \left (1-PTP_{h}\right) $	Rate of *Na*^+^/*Ca*^2+^ exchange

**Table 12:** List of model equations used in calculation of mitochondrial respiration equations (obtained from [11])

Equation	Biological significance
*A*_*res*_=(1.35*e*18)∗*NADHM*^0.5^/(*NAD*)^0.5^	*A*_*res*_ = affinity bracketed expression
$ V^{D}_{res} = exp\left (0.191 * PSI\right) $	Respiration potential generated
Proton pump equations	
*r*_1_=7*e*−7	Variable *r*_1_
*r*_2_=(2.54*e*−3)∗*A*_*res*_	Variable *r*_2_
$ r_{3} = 0.639 * V^{D}_{res} $	Variable *r*_3_
*r*_4_=7.58*e*13+(1.57*e*−4)∗*A*_*res*_	Variable *r*_4_
$ r_{5} = \left (1.73 + A_{res} * 1.06e-17\right) * V^{D}_{res} $	Variable *r*_5_
$ J^{H}_{res} = 360 * \rho _{res} * \left (\left (r1 + r2 - r3\right)/\left (r4 + r5\right)\right)$	Rate of transport through proton pump during respiration
Oxygen consumption rate equations	
*o*_1_=*A*_*res*_∗2.55*e*−3	Variable *o*_1_
*o*_2_=*A*_*res*_∗2.00*e*−5	Variable *o*_2_
$ o_{3} = 0.639*\left (V^{D}_{res}\right) $	Variable *o*_3_
$ o_{4} = \left (V^{D}_{res}\right) * A_{res} * 8.63e-18 $	Variable *o*_4_
*o*_5_=(1+*A*_*res*_∗2.08*e*−18)∗7.54*e*13	Variable *o*_5_
$ o_{6} = \left (1.73 + 1.06e-17 * A_{res}\right) * V^{D}_{res} $	Variable *o*_6_
*J*_*o*_=30∗*ρ*_*res*_∗(*o*1+*o*2−*o*3+*o*4)/(*o*5+*o*6)	Rate of oxygen consumption during respiration

**Table 13:** List of model equations used in calculation of mitochondrial Fo/F1-ATPase equations (obtained from [11])

Equation	Biological significance
*A*_*F*1_=(1.71*e*9)∗(*ATP*_*m*_)/(*ADP*_*mf*_∗*pim*)	*A*_*F*1_ = affinity bracketed expression
$ V^{D}_{F1} = exp(0.112 * PSI) $	ATPase potential generated
*F*_0_/*F*_1_ ATPase phosphorylation of *ADP*_*m*_	
*f*_1_=10.5∗*A*_*F*1_	Variable *f*_1_
$ f_{2} = 166 * V^{D}_{F1} $	Variable *f*_2_
$ f_{3} = (4.85e-12) * A_{F1} * V^{D}_{F1} $	Variable *f*_3_
*f*_4_=(1*e*7+0.135∗*A*_*F*1_)∗275	Variable *f*_4_
$ f_{5} = (7.74 + (6.65e-8) * A_{F1}) * V^{D}_{F1} $	Variable *f*_5_
*J*_*p*,*F*1_=−60∗*ρ*_*F*1_∗((*f*_1_−*f*_2_+*f*_3_)/(*f*_4_+*f*_5_))	Rate of *F*_0_/*F*_1_ ATPase phosphorylation
$ J_{H,F1} = -180 * \rho _{F1} * \left (0.213 + f_{1} - 169 * V^{D}_{F1}\right)/\left (f_{4} + f_{5}\right) $	Proton flux due to ATPase
*J*_*H*,*leak*_=*ρ*_*leak*_∗(*PSI*+24.6)	Mitochondrial membrane proton leak
*f*_*PDH*_=1/(1+(1.1∗(1+(15/(1+(*CAM*/0.05))^2^))))	Fraction of activated pyruvate
*J*_*red*_=*J*_*red*,*basal*_+6.3944∗*f*_*PDH*_∗*J*_*gly*,*total*_	NADH reduction rate
ATP/ADP antiport flux	
*ant*_1_=([*ATP*^4−^]_*i*_/[*ADP*^3−^]_*i*_)∗([*ADP*^3−^]_*m*_/[*ATP*^4−^]_*m*_)∗*exp*(−*PSI*/26.7)	Variable *ant*_1_
*ant*_2_=1+([*ATP*^4−^]_*i*_/[*ADP*^3−^]_*i*_)∗*exp*(−*PSI*/53.4)	Variable *ant*_2_
*ant*_3_=1+([*ADP*^3−^]_*m*_/[*ATP*^4−^]_*m*_)	Variable *ant*_3_
*J*_*ANT*_=*J*_*max*,*ANT*_∗((1−*ant*_1_)/(*ant*_2_∗*ant*_3_))	Rate of Adenine Nucleotide Translocator (ANT) activity
Phosphorylation of *ADP*_*m*_ from TCA cycle	
*J*_*p*,*TCA*_=(*J*_*red*,*basal*_/3)+0.84∗*f*_*PDH*_∗*J*_*gly*,*total*_	Unbound, 3- charged mitochondrial ADP concentration

**Table 14:** List of model equations used in calculation of cytosolic components (obtained from [11])

Equation	Biological significance
Glycolytic rate based on hexokinase	
*gly*_*num*_=(123.3∗(1+1.66∗*glc*)∗(*glc*∗*ATP*_*i*_))∗0.0249	Glycolytic rate numerator
*gly*_*denom*_=1+(4∗*ATP*_*i*_)+((1+2.83∗*ATP*_*i*_)∗1.3∗*glc*)+((1+2.66∗*ATP*_*i*_)∗0.16∗*glc*^2^)	Glycolytic rate denominator
*J*_*gly*,*total*_=*gly*_*num*_/*gly*_*denom*_	Glycolytic rate
*J*_*p*,*gly*_=2∗*J*_*gly*,*total*_	Phosphorylation of ADPi from glycolysis
*J*_*hyd*_=41∗(*ATP*_*i*_)+*J*_*hyd*,*max*_/(1+(8.7/*glc*)^2.7^)	Cytosolic hydrolysis of ATP

**Table 15:** List of model equations used in calculation of ER *Ca*^2+^ handling (modified from [21])

Equation	Biological significance
*J*_*er*,*out*_=(*V*_*IP*3_∗((*IP*_3_/(*IP*_3_+*d*_*IP*3_))^3^)∗((*CAC*/(*CAC*+*d*_*ACT*_))^3^)∗(*h*^3^)+*v*_*leak*_)∗(*CAER*−*CAC*)	*IP*_3_ receptor and leak
$ J_{serca} = V_{serca} * CAC^{2}/\left (k_{serca}^{2}+CAC^{2}\right) $	SERCA pump

**Table 16:** List of model equations used in *IP*_3_ step function (modified from [21])

Equation	Biological significance
*stepupf*=*heav*(*t*−*init*)	Heaviside step up function
*stepdownf*=*heav*(*t*−(*init*+*duration*))	Heaviside step down function
*IP*_3_=*baseline*+*amplitude*∗(*stepupf*−*stepdownf*)	*IP*_3_ step function

**Table 17:** List of model equations used in PTP Integration (modified from [21])

Equation	Biological significance
*τ*_*y*_=1000∗((1000/*cosh*(*CAM*/0.1))+0.1)	Time constant for secondary slow process
*τ*_*h*_=*τ*_*y*_/8	Time constant for PTP high conductance state
$ PTP_{h}^{\infty } = heav\left (y - y^{*}\right) $	Heaviside step function for PTP max value
*y*^*∞*^=*heav*(*CAM*−*CAM*^∗^)	Heaviside step function for y threshold value
*dy*/*dt*=(*y*^*∞*^−*y*)/*τ*_*y*_	Secondary slow process involved in opening of PTP high conductance state
$ dPTP_{h}/dt = \left (PTP_{h}^{\infty } - PTP_{h}\right)/\tau _{h} $	PTP high conductance state dynamics
$ J^{H}_{PTP} = perm_{l}^{H} * PTP_{l} * PSI * \left (H_{M} - 0.0000000398 * exp\left (-37.434 * PSI\right)/\left (1-exp\left (-37.434 * PSI\right)\right)\right) $	Proton flux through PTP in high conductance state
$ J^{Ca}_{PTP} = perm_{Ca} * PTP_{l} * J_{uni} * \left (1-postptp * PTP_{h}\right) $	Rate of *Ca*^2+^ ion transport across PTP
$ dH_{M}/dt = \left (f_{H_{M}}/\tau _{h}\right)*\left (J^{H}_{L} + J^{H}_{F1} - J^{H}_{res}+J^{H}_{PTP}\right) $	Change in mitochondrial proton concentration
*τ*_*l*_=*p*_6_+*amp*_*τ*_/*cosh*((*H*_*M*_−*p*_3_)/*p*_4_)	Time constant for PTP low conductance state
$ PTP_{l}^{\infty } = 0.5 * \left (1 + tanh\left (\left (p1 - H_{M}\right)/p_{2}\right)\right)$	Rate of change of polling function
$ dPTP_{l}/dt = \left (PTP_{l}^{\infty } - PTP_{l}\right)/\tau _{l} $	PTP low conductance state dynamics

## Results

### In the absence of pathology

**Fig. 3:**
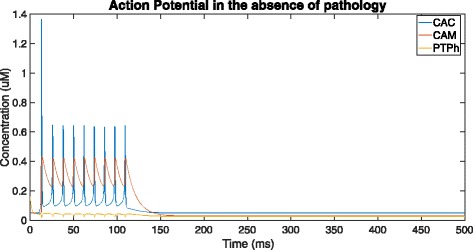
Overall time-course simulation showing cytosolic (CAC) and mitochondrial (CAM) Ca ^2+^ ion concentration along with tendency for opening of PTP in high-conductance state (PTPh) over a time of 500ms in the absence of *β*-amyloid deposition (by setting *V*_1_ = 0, *V*_*α*_ = 0 and *K*_1_ = 0)

**Fig. 4:**
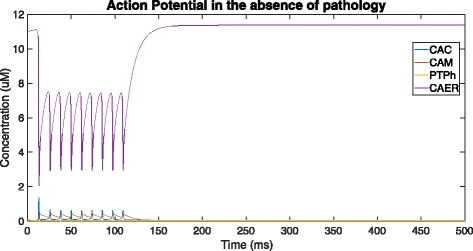
Addition of ER Ca ^2+^ ion concentration (CAER) to Fig. 3 and a visual representation of the scale of difference between the resting ER Ca ^2+^ level and cytosolic and mitochondrial resting Ca ^2+^ levels

Figures 3 and 4 show the time-course simulation of CAC, CAM and PTPh in a neuron stimulated by an action potential but devoid of deposition of misfolded *β*-amyloid oligomers. The *β*-amyloid concentration has been mathematically made null to depict the normal response of these parameters in a neuron to such a stimulus. In this scenario, there is an initial spike in cytosolic calcium level due to the action potential, which is subsequently followed by oscillations of lower amplitude in the ER, cytosol and mitochondria. The oscillations during such a stimulus are consistent with those observed in the Fall-Keizer model [7]. On adding mitochondria to *IP*_3_-mediated calcium ion release in isolation, long-term oscillations are observed (as detailed in [7]). However in our model, on incorporation of uniporter and PTP transport, there is transport of calcium ions outside this oscillatory mechanism, because of which the oscillations cannot be maintained and will stop. These oscillations are temporary, and die down after around 110 ms, restoring the calcium level in the ER, mitochondria and cytosol to their original values. There is naturally no stimulation of the mitochondrial PTP to open in the high conductance state due to the low resting concentration of Ca ^2+^ in the mitochondria.

In this case, the neuron responds as it would normally do to a stimulus, with the Ca ^2+^ ions being released rapidly from the ER via the *IP*_3_ channels to maintain calcium homeostasis across the cell. This sudden spurt results in the onset of oscillations of Ca ^2+^ ion concentration in the mitochondria, cytosol and ER. There is no further activity, and the concentration of Ca ^2+^ is sustained at a low equilibrium value.

### In the presence of pathology

**Fig. 5:**
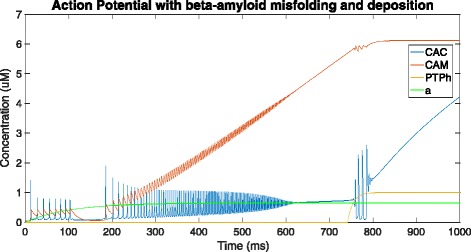
Graph depicting cytosolic (CAC) and mitochondrial (CAM) Ca ^2+^ ion concentration, *β*-amyloid concentration (a) and tendency for opening of PTP in high-conductance state (PTPh) over a time of 1000ms - in the presence of pathology (using the provided equations, parameter values and initial conditions in the XPP.ode file)

In Fig. 5, the neuron is affected by *β*-amyloid deposition, causing an increase in the entry of calcium ions into the cell and dysregulation of Ca ^2+^ channel receptors on the ER. At the start of the time-course simulation, an action potential is simulated causing the calcium levels in the cytosol to jump to over 1.4 *μ*M. Then, owing to Calcium-Induced Calcium Release (CICR), the process of sequestering and subsequent release of Ca ^2+^ ions, with the ER and mitochondria, the calcium level continues to oscillate for a while until it dies down and returns to its original level of around 0.05-0.1 *μ*M. As the amyloid concentration grows in the process, there is a sudden jump in the cytosolic and correspondingly mitochondrial calcium ion concentration due to the sudden release of a large volume of calcium ions from the ER (not shown in this graph). Amyloid metabolism affects the ryanodine and *IP*_3_ receptors located on the surface of the ER that regulate calcium release from the ER [20]. The sudden rise in calcium levels can be attributed to the ER sequestering a large proportion of the calcium that was initially in the cytosol and mitochondria.

As this continues, oscillation of calcium levels resumes, except this time the mitochondrial calcium ion concentration continuously increases, indicating an increase in sequestration of calcium ions by the mitochondria. As this constant rise is maintained for a period of time, as detailed in Oster et al. [8], a slower secondary process is activated, which results in the PTP opening in the high-conductance state. The opening of PTP in high-conductance state detailed by Oster et al. [8] follows exactly the same pattern, with the high, sustained levels of mitochondrial calcium ion concentration resulting in the activation of a slower, secondary process. This secondary slow process, on completion, results in the PTP opening in high conductance state.

**Fig. 6:**
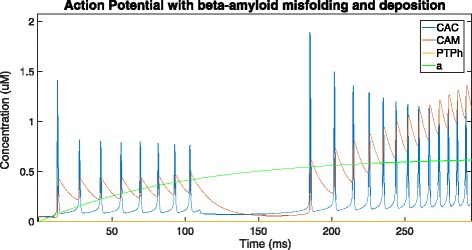
Zooming into 0-250 ms of Fig. 5 to visualise the oscillations and growth of amyloid concentration

The *β*-amyloid concentration rises slowly with the growth in cytosolic calcium, and this observation can be attributed to the relatively slow nature of amyloid processing to produce *β*-amyloids as compared to transfer of Ca ^2+^ ions (Fig. 6).

**Fig. 7:**
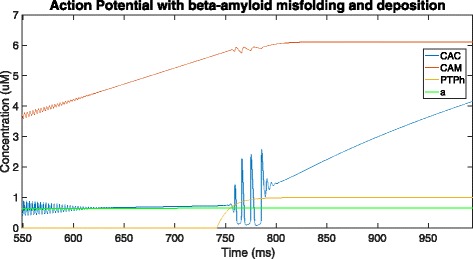
Zooming into 550-1000 ms of Fig. 5 to observe changes on opening of the PTP

Figure 7 shows that the PTPh starts opening at around 742 ms and takes 123 ms to reach the high-conductance state at around 865 ms. At the point of opening of the PTP in high conductance state (near 741 ms), mitochondrial Ca ^2+^ rushes out through the PTP and into the cytosol, which in turn is sequestered by the ER. The spurted release results in an oscillation of Ca ^2+^ ions between the ER and the cytosol, which dies down as the mitochondrial calcium level stagnates, indicating that the mitochondria is no longer functional.

When the PTPh opens in the high conductance state, calcium ions rush out of the mitochondria causing the CAM to level off. As the mitochondria stop functioning due to the PTP opening in high conductance, its calcium ion concentration is frozen, with CAC and CAER skyrocketing due to uncontrollable amounts of calcium ions flowing into the cell. This may be because the amyloid concentration has also increased over time (although in a much more regulated fashion), causing the plasma membrane to become more permeable and allowing more calcium ions into the neuron. Consequently, a jump occurs in cytosolic calcium ion concentration, which oscillates for a while and once stabilized begins to increase at an accelerated rate.

## Conclusion and discussion

This paper presents a mathematical model of the biological processes involved in the deposition of *β*-amyloids in and around the neurons and its effect on neuronal calcium signaling homeostasis. Using a detailed mitochondrial model of calcium signaling [7], the paper relates this change in calcium signaling in the cytosol to the calcium level in the mitochondrial matrix. Moreover, the introduction of the permeability transition pore and its characteristics allowed the model to depict the irreversible onset of apoptosis. By combining the models and features regarding *β*-amyloid deposition, mitochondrial calcium signaling and permeability transition pore activity, we simulated the opening of the permeability transition pore using amyloid deposition as the trigger. We found that, using the parameters and model equations above, high-conductance state is reached at around 865 ms after amyloid deposition begins.

The lack of comprehensive models of subcellular dynamics in Alzheimer’s disease poses a challenge for computational scientists to explore. We envisage a need for integrated modeling, i.e. selecting individual and specialized models and finding ways to combine them, to formulate a composite model of a neuron’s cell fate in AD.

In this paper, we have modeled only a single neuron undergoing mitochondrial PTP-related apoptosis, which is one of the mechanisms of cell death in AD. We have not studied necrosis due to Ca ^2+^ excitotoxicity, or looked at the effect of *β*-amyloid deposition and misfolding on cognitive functions, both of which can substantially extend the scope of this research. Furthermore, the effect of the neuron on its neighboring neurons in such a scenario has not been considered in this paper, as we assumed the neuron to exist in isolation. These issues are pertinent, and extending our composite model to address them could significantly improve our understanding of the disease.

In summary, we constructed a composite model by integrating three individual models to recapitulate a sequence of events and their repercussions that eventlead to neuronal death. Through this model, we are able to shed new light on a single sequence of processes starting from the deposition and misfolding of *β*-amyloid in and around the neuron all the way to neuronal apoptosis. Compared with existing models, this model provides a more comprehensive view of these molecular processes occurring in Alzheimer’s disease. It represents a step towards building more realistic models to facilitate the diagnosis and treatment of this aging-related disease.

## Additional file

Additional file 1 This file contains the parameters, initial conditions and equations used to generate the results in this paper. It can be run using XPP [19]. (ODE 8.18 kb)
